# Electronic cigarette use patterns and reasons for use among smokers recently diagnosed with cancer

**DOI:** 10.1002/cam4.1585

**Published:** 2018-06-14

**Authors:** Sara Kalkhoran, Gina R. Kruse, Nancy A. Rigotti, Julia Rabin, Jamie S. Ostroff, Elyse R. Park

**Affiliations:** ^1^ Tobacco Research and Treatment Center Department of Medicine Massachusetts General Hospital Boston MA USA; ^2^ Harvard Medical School Boston MA USA; ^3^ Mongan Institute for Health Policy Massachusetts General Hospital Boston MA USA; ^4^ Memorial Sloan Kettering Cancer Center New York NY USA

**Keywords:** electronic cigarettes, neoplasms, smoking, tobacco, tobacco use disorder

## Abstract

Many patients with cancer use electronic cigarettes (e‐cigarettes), yet little is known about patterns and reasons for use. Using cross‐sectional baseline data from a randomized controlled trial, we aimed to describe prevalence and correlates of e‐cigarette use, frequency of use, and reasons for use among smokers recently diagnosed with cancer. Participants (n = 302) included adults (age ≥18 years) recently diagnosed with varied cancer types who smoked ≥1 cigarette within the past 30‐d from two US academic medical centers. Participants reported ever and current e‐cigarette use, and current e‐cigarette users reported days of e‐cigarette use and the main reason for use. We compared current, former, and never e‐cigarette users by sociodemographics, cancer type, medical comorbidities, smoking behaviors, attitudes, and emotional symptoms, and described use among current e‐cigarette users. Of smokers recently diagnosed with cancer, 49% (n = 149) reported ever e‐cigarette use and 19% (n = 56) reported current use. Of current e‐cigarette users, 29% (n = 16) reported daily use. Current e‐cigarette users did not differ from former and never e‐cigarette users by cancer type, smoking behaviors, or emotional symptoms. Women were more likely to be current users than never users, and current e‐cigarette users had less education than former users. Most current e‐cigarette users reported using them to help quit smoking (75%). One in five smokers with cancer report current e‐cigarette use, but most are not using e‐cigarettes daily. The majority report using e‐cigarettes to quit smoking. E‐cigarette use by patients with cancer appears to reflect a desire to quit smoking.

## 1 INTRODUCTION

Electronic cigarette (e‐cigarette) use is prevalent among cigarette smokers in the US,^1^ including among those with medical comorbidities^2^ such as cancer.^3^ Smokers often report using e‐cigarettes to reduce or quit smoking or for health reasons,^4^, ^5^ but reasons for use specifically among patients with cancer are unknown. E‐cigarettes have not been associated with smoking cessation in the few observational studies of patients with cancer,^3^, ^6^ and while e‐cigarettes contain fewer carcinogens than conventional combustible cigarettes,^7^, ^8^, ^9^ their long‐term safety is unknown.

When assessing the potential risks and benefits of e‐cigarette use, it is important to understand how frequently e‐cigarette users are using these products. Experimentation with e‐cigarettes once or twice is likely to have different implications in terms of the effects on health and cigarette smoking behavior than regular daily use. There is great variability in how frequently current e‐cigarette users in the general adult US population report using e‐cigarettes. A recent study found that among the 5.5% of US adults reporting current e‐cigarette use, 42% reported use on 2 or fewer days in the past 30 days and 21% reported daily e‐cigarette use.^10^ While past studies have assessed prevalence of e‐cigarette use among smokers with cancer, there have been few estimates of frequency of or reasons for e‐cigarette use in this population.

Given that cessation improves survival and other health outcomes in smokers with cancer,^11^, ^12^ a cancer diagnosis provides a critical opportunity to promote smoking behavior change.^13^ To best counsel and assist cancer patients who smokers with smoking cessation, it is necessary to understand their use of other nicotine and tobacco products such as e‐cigarettes. We aimed to describe, among smokers recently diagnosed with cancer: (1) prevalence and correlates of e‐cigarette use, (2) patterns of e‐cigarette use, and (3) reasons for e‐cigarette use.

## 2 METHODS

We conducted a secondary cross‐sectional analysis of baseline enrollment data from the Smokefree Support Study, a multi‐site, randomized controlled comparative effectiveness trial of tobacco treatment interventions in smokers with recent diagnosis of various tobacco and nontobacco‐related cancers. The trial protocol has been described previously.^14^ Enrollment took place from November 2013 through June 2017. The MGH/Partners Healthcare Institutional Review Board approved this study.

### 2.1 Study population

Participants included adults (age ≥18 years) recently diagnosed with cancer (within approximately 3 months or 4 visits of initial visit with an oncologist at one of the study sites) who smoked a cigarette, even a puff, in the past 30 days. Recruitment occurred from two academic medical centers: Partners Healthcare (Massachusetts General Hospital and Dana Farber Cancer Institute, Boston, MA) and Memorial Sloan Kettering Cancer Center (New York, NY). Other inclusion and exclusion criteria were described previously.^14^

### 2.2 Measures

Following consent and prior to randomization, participants completed a baseline assessment to assess sociodemographics, tobacco‐related behaviors and attitudes, and physical and emotional symptoms.

#### 2.2.1 E‐cigarette use

We asked participants about ever and current e‐cigarette use (within the past 30 days). We categorized e‐cigarette use into 3 categories: current e‐cigarette use, former e‐cigarette use (ever use of e‐cigarettes, but not within the past 30 days), and never e‐cigarette use. Current e‐cigarette users also reported number of days of e‐cigarette use in the past 30 days and the main reason for e‐cigarette use, which was asked as a multiple choice question with the following response options: help to quit smoking cigarettes, have something to use in a nonsmoking area, use as a less risky product long‐term, or other.

#### 2.2.2 Covariates

Demographic and socioeconomic covariates included age, sex, race/ethnicity, education, marital/partner status, and insurance. Participants also self‐reported medical comorbidities (asthma, chronic obstructive pulmonary disease/emphysema, diabetes, heart attack, hypertension, stroke). We obtained information on cancer type from medical records, and categorized cancer types into smoking‐related (lung, esophageal, head and neck, bladder, kidney, liver, pancreatic, colorectal, anal, small intestinal, gastric, or cervical)^15^ and nonsmoking‐related (prostate, testicular, penile, breast, lymphoma, melanoma, or noncervical gynecologic cancer).

Smoking‐related characteristics included number of cigarettes smoked per day, past quit attempts, past or current use of smoking cessation medications, and nicotine dependence measured as first cigarette within 30 minutes of waking.^16^ We defined participants who reported that “no one is allowed to smoke anywhere” in their homes as having a home smoking ban.

For perceived benefits of quitting smoking, we asked participants the following questions on a scale of 0‐10 (0 being not at all and 10 being very much): How much would quitting smoking reduce your chances of complications from treatment, improve your chances of getting the full benefit from treatment, reduce your chances of developing cancer again, and reduce your chances of developing other health problems.^14^ We created a composite score from the sum of these 5 variables (range 0‐50). For participants with missing data for 1 of the 5 questions, the score was imputed as the mean of the responses to the other 4 questions. Intention to quit/stay quit was measured on a 10‐point scale from 0 (“I enjoy smoking so much I will never consider quitting no matter what happens”) to 9 (“I have quit and I am 100% confident that I will never smoke again”).^17^ Importance of quitting was asked on a scale of 0 (not at all important) to 10 (very important). Urges to smoke in the past 24 hours were reported on a scale 0 (not at all) to 5 (all the time). Distress over the past 2 weeks was measured on a scale from 0 (no distress) to 10 (extreme distress).

#### 2.2.3 Analysis

Of the 303 study participants, we excluded 1 participant with missing data on e‐cigarette use from analyses for a total study sample size of 302. We compared the sociodemographic, smoking‐related, cancer‐related, and physical and emotional symptoms among never, former, and current e‐cigarette users using chi‐square tests and two‐sided *t* tests. We defined statistical significance as a *P*‐value <.05. We also described frequencies of days of e‐cigarette use and reasons for e‐cigarette use among current e‐cigarette users. We used Stata version 14 for all analyses.

## 3 RESULTS

Of the 302 study participants, 149 (49%) reported ever e‐cigarette use and 56 (19%) reported current e‐cigarette use. Current e‐cigarette users were more likely to be female than never e‐cigarette users, and had lower education than former e‐cigarette users (Table 1). Current e‐cigarette users did not differ significantly from former and never e‐cigarette users by smoking history, medical history, perceived benefits of quitting smoking, or physical or emotional symptoms (Tables 1, 2, 3).

**Table 1:** Participant sociodemographic and medical characteristics by e‐cigarette use status

	Current e‐cigarette use (n = 56)	Former e‐cigarette use (n = 93)	*P*‐value (vs current use)	Never e‐cigarette use (n = 153)	*P*‐value (vs current use)
Age—mean (SD)	56.8 (10.2)	57.7 (10.1)	.61	59.2 (9.3)	.11
Sex—n(%)					
Female	39 (69.6)	51 (54.8)	.07	79 (51.6)	.02
Male	17 (30.4)	42 (45.2)		74 (48.4)	
Race^a^—n(%)					
White	51 (91.1)	76 (81.7)	.28	127 (83.0)	.35
Black	3 (5.4)	12 (12.9)		15 (9.8)	
Other	2 (3.6)	5 (5.4)		11 (7.2)	
Hispanic ethnicity^a^	2 (3.6)	5 (5.5)	.60	5 (3.3)	.93
Education^a^—n(%)					
High school graduate or less	24 (42.9)	21 (23.3)	.03	48 (32.4)	.16
Some college/vocational school	22 (39.3)	41 (45.6)		55 (37.2)	
College graduate or more	10 (17.9)	28 (31.1)		45 (30.4)	
Marital status^a^—n(%)					
Married/living as married	32 (57.1)	47 (52.8)	.69	85 (57.1)	.83
Single, never married	7 (12.5)	18 (20.2)		13 (8.7)	
Divorced or separated	14 (25.0)	20 (22.5)		40 (26.9)	
Widowed	3 (5.4)	4 (4.5)		11 (7.4)	
Types of insurance^a^^,^ b—n(%)					
Employer‐sponsored/Military	28 (50.0)	38 (42.7)	.30	64 (43.2)	.84
Medicaid	4 (7.1)	15 (16.9)		18 (12.2)	
Medicare	13 (23.2)	22 (24.7)		36 (24.3)	
Individual (self‐purchased)	7 (12.5)	12 (13.5)		18 (12.2)	
Other	4 (7.1)	2 (2.3)		12 (8.1)	
Number of medical comorbidities—n(%)					
0	18 (32.1)	38 (40.9)	.06	69 (45.1)	.19
1	25 (44.6)	24 (25.8)		50 (32.7)	
≥2	13 (23.2)	31 (33.3)		34 (22.2)	
Smoking‐related cancer—n(%)	38 (67.9)	54 (58.1)	.23	88 (57.5)	.18
Cancer diagnosis—n(%)					
Lung	19 (33.9)	23 (24.7)	.90	45 (29.4)	.96
Head and neck	8 (14.3)	7 (7.5)		16 (10.5)	
Esophageal	2 (3.6)	3 (3.2)		2 (1.3)	
Bladder	2 (3.6)	7 (7.5)		9 (5.9)	
Kidney	1 (1.8)	3 (3.2)		4 (2.6)	
Liver	1 (1.8)	3 (3.2)		3 (2.0)	
Pancreatic	2 (3.6)	2 (2.2)		2 (1.3)	
Colorectal	2 (3.6)	3 (3.2)		3 (2.0)	
Anal	1 (1.8)	1 (1.1)		2 (1.3)	
Small intestinal	0 (0)	1 (1.1)		0 (0)	
Gastric	0 (0)	0 (0)		1 (0.7)	
Cervical	0 (0)	1 (1.1)		1 (0.7)	
Prostate	2 (3.6)	5 (5.4)		16 (10.5)	
Testicular	0 (0)	1 (1.1)		0 (0)	
Breast	12 (21.4)	28 (30.1)		37 (24.2)	
Lymphoma	2 (3.6)	3 (3.2)		4 (2.6)	
Melanoma	1 (1.8)	2 (2.2)		3 (2.0)	
Noncervical gynecologic cancer	1 (1.8)	0 (0)		4 (2.6)	
Penile	0 (0)	0 (0)		1 (0.7)	

a Missing data were as follows: 5 for Hispanic ethnicity, 8 for education, 8 for marital status, 9 for insurance.

b Options were not mutually exclusive and therefore percentages may not add up to 100%.

**Table 2:** Tobacco use behaviors and attitudes by e‐cigarette use status

	Current e‐cigarette use (n = 56)	Former e‐cigarette use (n = 93)	*P*‐value (vs current use)	Never e‐cigarette use (n = 153)	*P*‐value (vs current use)
Cigarettes/d^a^					
1‐5	7 (13.0)	14 (15.1)	.47	39 (25.8)	.28
6‐10	17 (31.5)	25 (26.9)		43 (28.5)	
11‐15	7 (13.0)	21 (22.6)		20 (13.3)	
15‐20	17 (31.5)	28 (30.1)		31 (20.5)	
>20	6 (11.1)	5 (5.4)		18 (11.9)	
First cigarette within 30 min of waking^a^—n(%)	43 (76.8)	70 (77.8)	.89	100 (66.7)	.16
Any past quit attempt—n(%)	48 (85.7)	88 (94.6)	.06	137 (89.5)	.44
Use of smoking cessation medications^a^—n(%)					
Ever use	45 (80.4)	82 (89.1)	.14	111 (73.0)	.28
Current use	10 (17.9)	17 (18.3)	.95	31 (20.3)	.70
Home smoking ban^a^—n(%)	23 (42.6)	41 (44.6)	.82	81 (54.4)	.14

a Missing data were as follows: 4 for cigarettes per day, 6 for time to first cigarette, 2 for ever use of smoking cessation medication, 7 for home smoking ban.

**Table 3:** Behavioral, physical, and psychological characteristics by e‐cigarette use status

	Current e‐cigarette use (n = 56)	Former e‐cigarette use (n = 93)	*P*‐value (vs current use)	Never e‐cigarette use (n = 153)	*P*‐value (vs current use)
Perceived benefits of quitting smoking (range 0‐50)—mean (SD)	43.7 (8.2)	45.6 (6.4)	.14	43.0 (8.7)	.61
Importance of quitting (range 0‐10)^a^—mean (SD)	9.2 (1.5)	9.5 (1.3)	.17	9.2 (1.8)	.87
Intention to quit/stay quit (range 0‐9)^a^—mean (SD)	6.1 (1.7)	6.1 (1.7)	.90	5.7 (1.8)	.16
Urges to smoke (range 0‐5)^a^—mean (SD)	2.6 (1.3)	2.6 (1.4)	.91	2.5 (1.2)	.71
Distress score (range 0‐10)^a^—mean (SD)	6.8 (3.0)	7.4 (2.6)	.17	6.8 (2.8)	.98

a Missing data were as follows: 20 for perceived benefits of quitting, 10 for importance of quitting, 28 for intention to quit/stay quit, 12 for urges to smoke, 10 for distress score.

**Figure 1:**
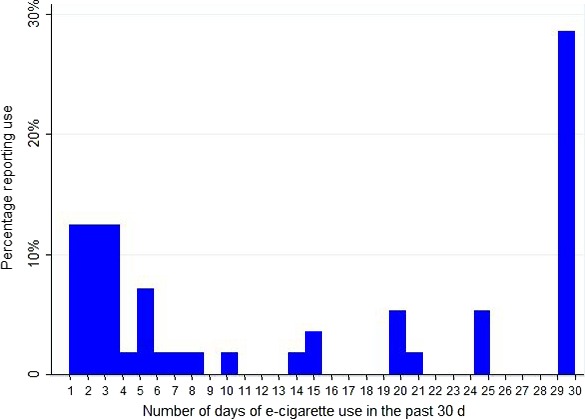
Number of days of e‐cigarette use in the past 30‐d among smokers who reported current use of e‐cigarettes

Among current e‐cigarette users (n = 56), the mean number of days of e‐cigarette use in the past 30 days was 14. Sixteen current e‐cigarette users reported daily e‐cigarette use and 26 reported 5 or fewer days of e‐cigarette use in the past 30 days (Figure 1). In terms of the relationship between cigarette smoking and e‐cigarette use, 13% (n = 2) of participants who reported daily e‐cigarette use reported smoking 5 or fewer cigarettes per day. Similarly, 12% (n = 3) of participants reporting 5 or fewer days of e‐cigarette use in the past 30 days reported smoking 5 or fewer cigarettes per day. The percentage of smokers reporting smoking 16 or more cigarettes per day was 20% (n = 3) for those reporting daily e‐cigarette use and 8% (n = 2) for those reporting 5 or fewer days of e‐cigarette use in the past 30 days.

**Figure 2:**
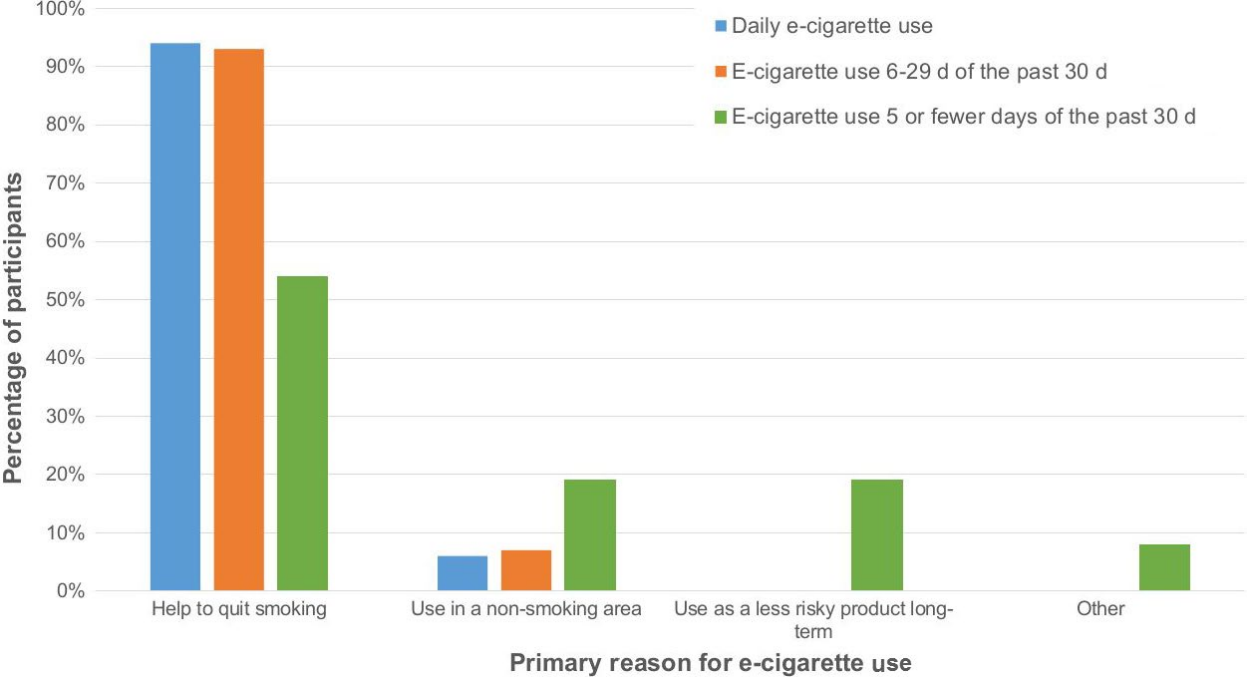
Reasons for e‐cigarette use by days of e‐cigarette use among smokers who reported current use of e‐cigarettes

The most common reason given for e‐cigarette use among all current users was to help quit cigarettes (n = 42), followed by use in a nonsmoking area (n = 7), use as a less risky product long‐term (n = 5), and other (n = 2). Of the 30 current e‐cigarette users who reported using e‐cigarettes on 6 or more days of the past 30 days, most (n = 28) selected “help to quit smoking cigarettes” as their primary reason for using e‐cigarettes (Table 2). In contrast, among the 26 smokers reporting using e‐cigarettes on 5 or fewer days of the past 30 days, approximately half (n = 14) selected to help quit smoking as the primary reason for e‐cigarette use (*P* = .005).

## 4 DISCUSSION

We found that nearly one‐fifth of cigarette smokers recently diagnosed with various cancers reported currently using e‐cigarettes. This estimate is similar to that from a clinic‐based study that showed 25% of cancer patients who reported current smoking also reported past 30‐d (current) e‐cigarette use,^3^ and one that showed 22% of current and former tobacco users with head and neck cancers had used e‐cigarettes as part of a quit attempt.^6^ Notably, these e‐cigarette use rates are higher than the 16% reported among smokers in the general US adult population.^1^ Our estimates are also higher than those from a study of cancer survivors using data from the 2014 National Health Interview Survey, which found that 34.3% of current cigarette smokers reported ever using e‐cigarettes, and 15.6% of current cigarette smokers reported current e‐cigarette use.^18^ Since most current e‐cigarette users in this study reported that their main reason for using e‐cigarettes was to help quit smoking, one factor that may be underlying the high e‐cigarette prevalence is increased motivation to change smoking behaviors at the time of a new cancer diagnosis. Asking about current e‐cigarette use may also help oncology providers to encourage their tobacco dependent patients to seek evidence‐based tobacco treatment.

In terms of sociodemographic factors, we found that a higher percentage of smokers who reported current e‐cigarette use were women compared to smokers who reported never using e‐cigarettes. Neither of the previous studies on cancer patients and e‐cigarette use^3^, ^6^ found differences in sex and education between e‐cigarette users and nonusers, but these studies did not compare current and never e‐cigarette users directly. Further studies should assess the association between sex and e‐cigarette initiation and continued use among smokers with cancer. We also found that smokers who currently used e‐cigarettes had lower levels of education than smokers who were former e‐cigarette users. One potential explanation is that smokers with cancer who have lower levels of education are more likely to continue e‐cigarette use after trying it. Alternatively, since we don't know how long current e‐cigarette users have been using the products, it may be that smokers with lower education are just recently trying e‐cigarettes for the first time while smokers with higher education tried e‐cigarettes previously. Few studies have compared current and former e‐cigarette users,^5^, ^19^ but studies comparing these groups can be helpful in identifying factors potentially associated with persistent e‐cigarette use.

A prior study of smokers with various cancers found that smokers who reported current use of e‐cigarettes were more nicotine dependent and smoked more cigarettes per day compared to smokers who were not currently using e‐cigarettes.^3^ However, we did not find statistically significant differences between current, former, and never e‐cigarette users in terms of daily cigarette consumption and nicotine dependence, which may be attributable to the smaller sample size of this study. Although not statistically different, we did find that current e‐cigarette users reported a greater number of cigarettes per day than former or never e‐cigarette users, and found higher nicotine dependence among current e‐cigarette users compared to never e‐cigarette users. This suggests that smokers with cancer who use e‐cigarettes have smoking characteristics that make successful smoking cessation more challenging, and e‐cigarette use in this population may represent an attempt to face these challenges.

Approximately 30% of current e‐cigarette users in this study reported using e‐cigarettes daily. This percentage is slightly higher than the 21% of current e‐cigarette users in the general population reporting daily use.^10^ Smokers who reported more frequent use of e‐cigarettes cited using e‐cigarettes to help to quit smoking more often than smokers reporting infrequent (ie 5 or fewer days in the past month) e‐cigarette use. A study using data from the Population Assessment of Tobacco and Health Survey from 2013 to 2014 found that a large percentage of daily e‐cigarette users reported using e‐cigarettes to cut down on cigarette use (89%) compared to those who used e‐cigarettes less on 2 or fewer days of the past 30‐days (58%).^10^ While the e‐cigarette market has expanded between 2013 and 2017, when some of the data from the present study were collected, these findings together suggest that more frequent use among current users may be associated with greater intent for smoking behavior change. Longitudinal studies are needed to evaluate the relationship between frequency of e‐cigarette use and smoking cessation in cancer patients. We also found that more former e‐cigarette users reported having made any quit attempt in the past compared to current e‐cigarette users. While former e‐cigarette users were not asked about their reasons for e‐cigarette use, and the timing of e‐cigarette use with respect to past quit attempts is unknown, this may also be suggestive of an association between e‐cigarette and quitting‐related intentions and behaviors that should be explored in future studies.

The finding that the majority of current e‐cigarette users in this study reported the reason for using e‐cigarettes was to quit smoking cigarettes is consistent with other clinical populations of smokers.^5^ It is important that providers caring for newly diagnosed cancer patients are aware of the high prevalence of and reasons for e‐cigarette use among smokers, as patients may come to their providers with questions about these products.^20^ Patients should be advised of the unknown long‐term health risks of e‐cigarettes and their unknown efficacy for smoking cessation. Patients should be encouraged to use evidence‐based medication and counseling for smoking cessation as recommended in a 2015 joint statement by the American Association for Cancer Research and American Society of Clinical Oncology.^21^ Providers should assess for current e‐cigarette use and consider all sources of nicotine that a patient is using when providing recommendations for use and dosing of FDA‐approved cessation medications such as nicotine replacement therapy.

This study has several limitations of note. Data were collected from smokers participating in a smoking cessation trial being conducted by two academic medical centers in the northeastern US and may not be generalizable to other cancer patient populations. The analyses examining the relationship between various patterns of cigarette smoking and patterns of e‐cigarette use were based on a small number of patients. We do not have data on e‐cigarette use among patients with cancer who do not smoke cigarettes and cannot assess extent of any e‐cigarette use in this population. Data on cigarette and e‐cigarette use were collected by participant self‐report and may be subject to reporting bias. We are unable to determine the timing of ever and current e‐cigarette use with respect to time of cancer diagnosis, the duration of e‐cigarette use among current and former users, or whether initial e‐cigarette use was within the past 30 days, which would affect our interpretation of reported frequency of e‐cigarette use. Additionally, former e‐cigarette users may represent a heterogenous group in terms of frequency and duration of use. Finally, since our study focuses on current smokers within 3 months of an initial visit with an oncologist, it does not capture individuals who quit smoking after diagnosis but more than 30 days ago, and these individuals may differ in their e‐cigarette use patterns and reasons for use.

Half of smokers recently diagnosed with cancer report ever use of e‐cigarettes. Nearly one in five reported current use of e‐cigarettes, and they were more likely to be women. Most patients with cancer reported using e‐cigarettes as a quitting strategy. Desire to quit cigarettes may influence e‐cigarette use in this population, and future longitudinal research is needed to determine whether e‐cigarette use deters or facilitates smoking abstinence.

## CONFLICT OF INTEREST

Drs. Kalkhoran, Rigotti, Ostroff, and Park receive royalties from UpToDate. Dr. Ostroff also receives research support from the CVS Foundation. Dr. Kruse has a financial interest in Dimagi, Inc. and is a paid consultant for Click Therapeutics.
